# Epidemiological study of scarlet fever in Shenyang, China

**DOI:** 10.1186/s12879-019-4705-9

**Published:** 2019-12-21

**Authors:** Huijie Chen, Ye Chen, Baijun Sun, Lihai Wen, Xiangdong An

**Affiliations:** Department of Infectious Disease, Shenyang Health Service and Administrative Law Enforcement Center (Shenyang Center for Disease Control and Prevention), Shenyang, 110031 China

**Keywords:** Scarlet fever, Spatiotemporal epidemiological characteristics, Shenyang

## Abstract

**Background:**

Since 2011, there has been an increase in the incidence of scarlet fever across China. The main objective of this study was to depict the spatiotemporal epidemiological characteristics of the incidence of scarlet fever in Shenyang, China, in 2018 so as to provide the scientific basis for effective strategies of scarlet control and prevention.

**Methods:**

Excel 2010 was used to demonstrate the temporal distribution at the month level and ArcGIS10.3 was used to demonstrate the spatial distribution at the district/county level. Moran’s autocorrelation coefficient was used to examine the spatial autocorrelation and the Getis-Ord statistic was used to determine the hot-spot areas of scarlet fever.

**Results:**

A total of 2314 scarlet fever cases were reported in Shenyang in 2018 with an annual incidence of 31.24 per 100,000. The incidence among males was higher than that among females(*p*<0.001). A vast majority of the cases (96.89%) were among children aged 3 to 11 years. The highest incidence was 625.34/100,000 in children aged 5–9 years. In 2018 there were two seasonal peaks of scarlet fever in June (summer-peak) and December (winter-peak). The incidence of scarlet fever in urban areas was significantly higher than that in rural areas(*p*<0.001). The incidence of scarlet fever was randomly distributed in Shenyang. There are hotspot areas located in seven districts.

**Conclusions:**

Urban areas are the hot spots of scarlet fever and joint prevention and control measures between districts should be applied. Children aged 3–11 are the main source of scarlet fever and therefore the introduction of prevention and control into kindergarten and primary schools may be key to the control of scarlet fever epidemics.

## Background

Scarlet fever is caused by erythogenic toxins produced by group A Streptococcus (GAS) which is mainly transmitted through direct contact with saliva and nasal fluids from infected persons [1]. The disease is characterized by a sorethroat, fever, and a sandpaper-like rash on reddened skin and most commonly occurs in winter and spring and most commonly affects children [2].

In 2011, an outbreak of scarlet fever hit Hong Kong (China) and over 600 cases were reported by the end of June 2011 [3], with two deaths. The same year in April to July, Shanghai witnessed an unprecedented outbreak of scarlet fever among children. In recent years, the number of scarlet fever cases have been increasing in China [4]. In 2017, a total of 74,369 cases of scarlet fever was reported, compared with 34,207 in 2013 and 54,247 in 2014 and 68,249 in 2015 and 59,282 in 2016 respectively [5].

As a useful tool, geographic information system (GIS) has been widely applied in infectious diseases surveillance [6–8]. However, few studies [9, 10] have focused on the spatiotemporal characteristics of scarlet fever. In Shenyang, some researchers described the epidemiology of scarlet fever [11–14], but none explored the spatiotemporal patterns.

In our study, we used Excel 2010 and ArcGIS 10.3 to depict the spatiotemporal characteristics of scarlet fever in Shenyang followed the methods of Qi Zhang et al., 2017 [9]. The objective of our study was to describe the temporal and spatial epidemic characteristics of scarlet fever in Shenyang and explore the socio-demographic and geographic risk factors affecting the epidemic of scarlet fever in order to provide scientific basis for the prevention and control measures of scarlet fever in Shenyang.

## Methods

### Study area

Shenyang is the capital city of Liaoning province. It is a prefecture-level city in China, including both urban and rural areas. Shenyang is located in latitude 41°11′–43°02′N and longitude 122°25′–123°48′E, measures 12,860 Sq km and consists of 13 districts and 214 towns [15]. The districts are named as follows:(1) Heping, (2) Shenhe, (3) Dadong, (4) Huanggu, (5) Tiexi, (6) Sujiatun, (7) Hunnan, (8) Shenbeixin, (9) Yuhong, (10) Liaozhong, (11) Kangping, (12) Faku, (13) Xinmin. Among them Heping, Shenhe, Dadong, Huanggu, Tiexi, Sujiatun, Hunnan, Shenbeixin and Yuhong belong to urban areas. Liaozhong, Kangping, Faku and Xinmin belong to rural areas. The population in Shenyang was 7,408,238 in 2018.

### Data sources

All reported cases (including the patient’s age, sex, occupation, and address) of scarlet fever in 2018 were extracted from the Nationwide Notifiable Infectious Diseases Reporting Information System (NIDRIS) which were used under license and not publicly available. Additionally, we retrieved population data from the Official Website of Shenyang Statistical Bureau. Maps of Shenyang were downloaded from Data Sharing Infrastructure of Earth System Science (http://www.geodata.cn/).

### Case definition

The diagnosis of scarlet fever is based on the clinical criteria established by the Law of Communicable Diseases Prevention and Control of the People’s Republic China and Guidance offered by the Chinese Ministry of Health [16]. The clinical manifestations of scarlet fever (ICD A38.01) are acute onset of fever, pharyngitis with “strawberry tongue” which is a tongue with a whitish coat through which the enlarged fungiform papillae project as red points resembling a strawberry, red rash with a sandpaper feel, and itching, as well as a throat swab culture and stain and a skin smear stain to confirm Group A Streptococcus (GAS) infection. Scarlet fever is a notifiable Group B infectious disease according to the China National Notifiable Infectious Disease Surveillance System (NNIDSS) [17]. The clinical criteria [16] is widely used by Chinese doctors.

### Statistical analysis

SPSS 23.0 software was used for statistical analysis. Normal distribution measurements were expressed by mean (x) and standard deviation (s), t-test was used for comparison between the two groups and analysis of variance (ANOVA) was used for comparison between groups. Count data were expressed by incidence rate,chi-squared test was used for comparison between groups. The significance level used was *p*<0.05.

### Spatial autocorrelation analysis

The spatial autocorrelation (Global Moran’s I) statistic measure was used to evaluate whether the disease patterns are clustered, dispersed or randomly distributed in the area. In general, positive spatial autocorrelation occurs when Moran’s I index values close to + 1.0, this means similar values cluster together namely clustered, whereas negative spatial autocorrelation occurs when Moran’s I index values close to − 1.0, this means dissimilar values cluster together namely dispersed. Moran’s I index values close to zero indicate no autocorrelation, this means randomly distributed in the study area [18] . ArcGIS 10.3 was used to perform the analysis.

### Hotspot analysis

The hotspot analysis (Getis-Ord, Gi*)statistic measure, was used to evaluate the intensity and stability of spatial clusters and it has the advantage of distinguishing high-high value clusters (hotspot) or low-low value clusters (coldspot). If the Z (Gi*) score is positive and significant, it shows that one district and its neighbouring regions have a relatively high frequency of scarlet fever incidents, which is a hotspot. On the contrary, if the Z (Gi*) score is negative and significant, it shows that one district and its neighbouring regions have a relatively low frequency of scarlet fever incidents, which is a coldspot. In general, districts with Z-scores > 2.58 or Z-scores <− 2.58 were considered to be significant at 99% confidence level (*p* < 0.01). Districts with Z-scores between 1.96–2.58 or Z-scores between − 1.96 – − 2.58 were considered to be significant at 95% confidence level (*p* < 0.05). Districts with Z-scores between 1.65–1.96 or Z-scores between − 1.65 – − 1.96 were considered to be significant at 90% confidence level (*p* < 0.10) [19]. ArcGIS 10.3 was used to perform the analysis.

## Results

### Demographic characteristics

A total of 2422 scarlet fever cases were reported in 2018 and 108 of these cases were excluded because they did not reside in Shenyang. Finally, a total of 2314 cases were included in the analysis. The annual incidence rate of scarlet fever was 31.24 per 100,000 population. The incidence in males was significantly higher than that in females(*p*<0.001). There was a significant difference in the incidence among groups(*p*<0.001) and the incidence of 5–9 years group was the highest (Table 1).

**Table 1:** Incidence of scarlet fever in different group in Shenyang in 2018

Group	Cases	Population	Incidence
Gender			
Male	1375	3,651,661	37.65
Female	939	3,756,577	25.00
X^2^			95.013
P			<0.001
Age group (Years)			
0–4	382	299,215	127.67
5–9	1772	283,368	625.34
10–14	130	253,991	51.18
≥ 15	30	6,571,664	0.46
X^2^			34,947.179
P			<0.001

Note: Incidence is the average annual incidence (per 100,000 population per year)

Population is the average population of Shenyang in 2018

### Temporal pattern

**Fig. 1:**
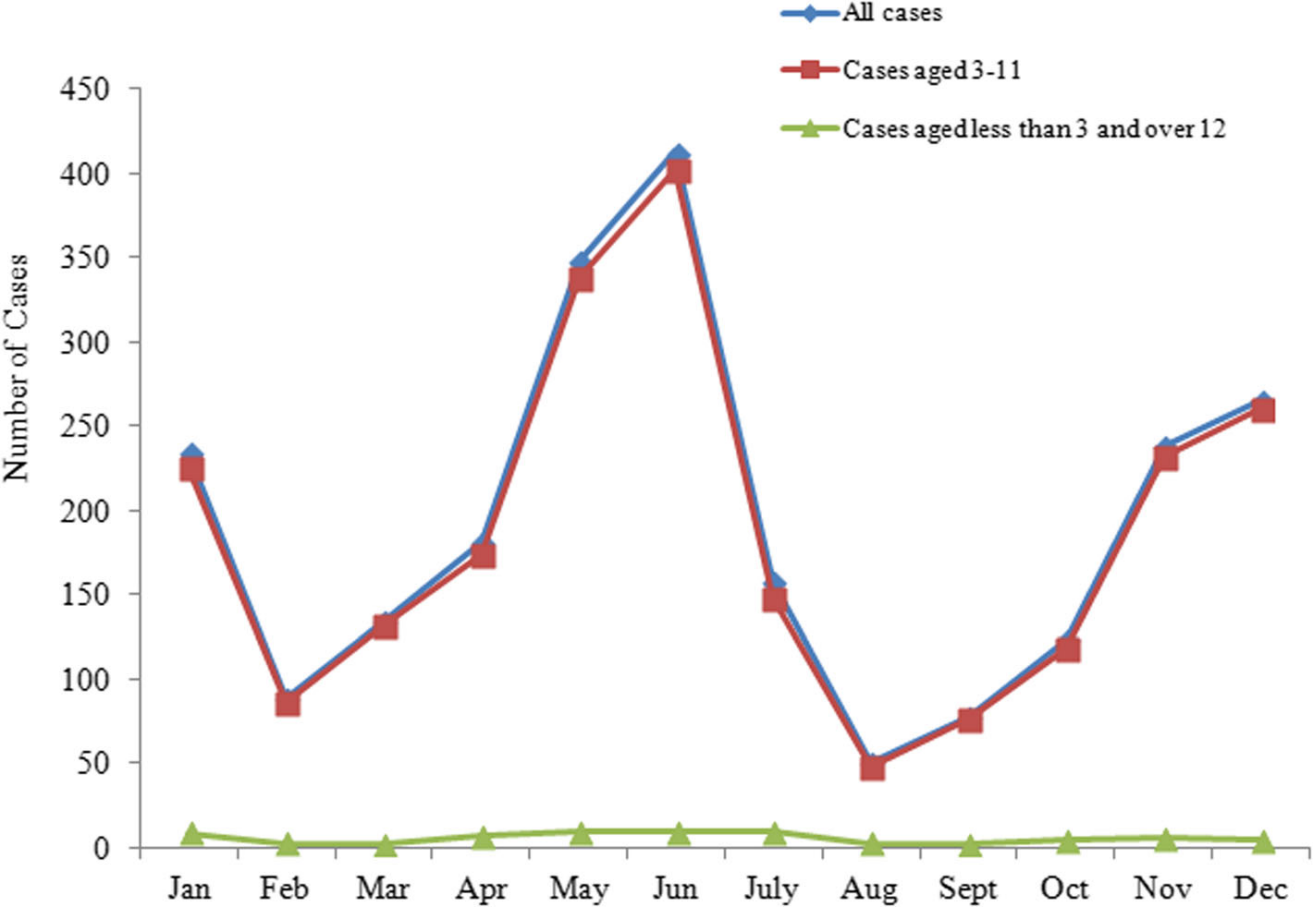
Monthly case of scarlet fever in Shenyang in 2018. Each point represents the number of scarlet fever cases in a specific month. All of the points are lined to indiate the trend of the scarlet cases in different groups. Different colors represent different groups

The monthly distribution of scarlet fever cases had obvious seasonality in Shenyang through graphical observation. The first large peak occurred in June (Summer-peak), followed by a small peak in December (Winter-peak). Children aged 3–11 accounted for 96.89% of all scarlet fever cases. A seasonal trend was observed in the group of children aged 3–11 but was not observed in cases outside of this age bracket, although this represented only 3.11% of the population sampled (Fig. 1).

### Spatial pattern

**Fig. 2:**
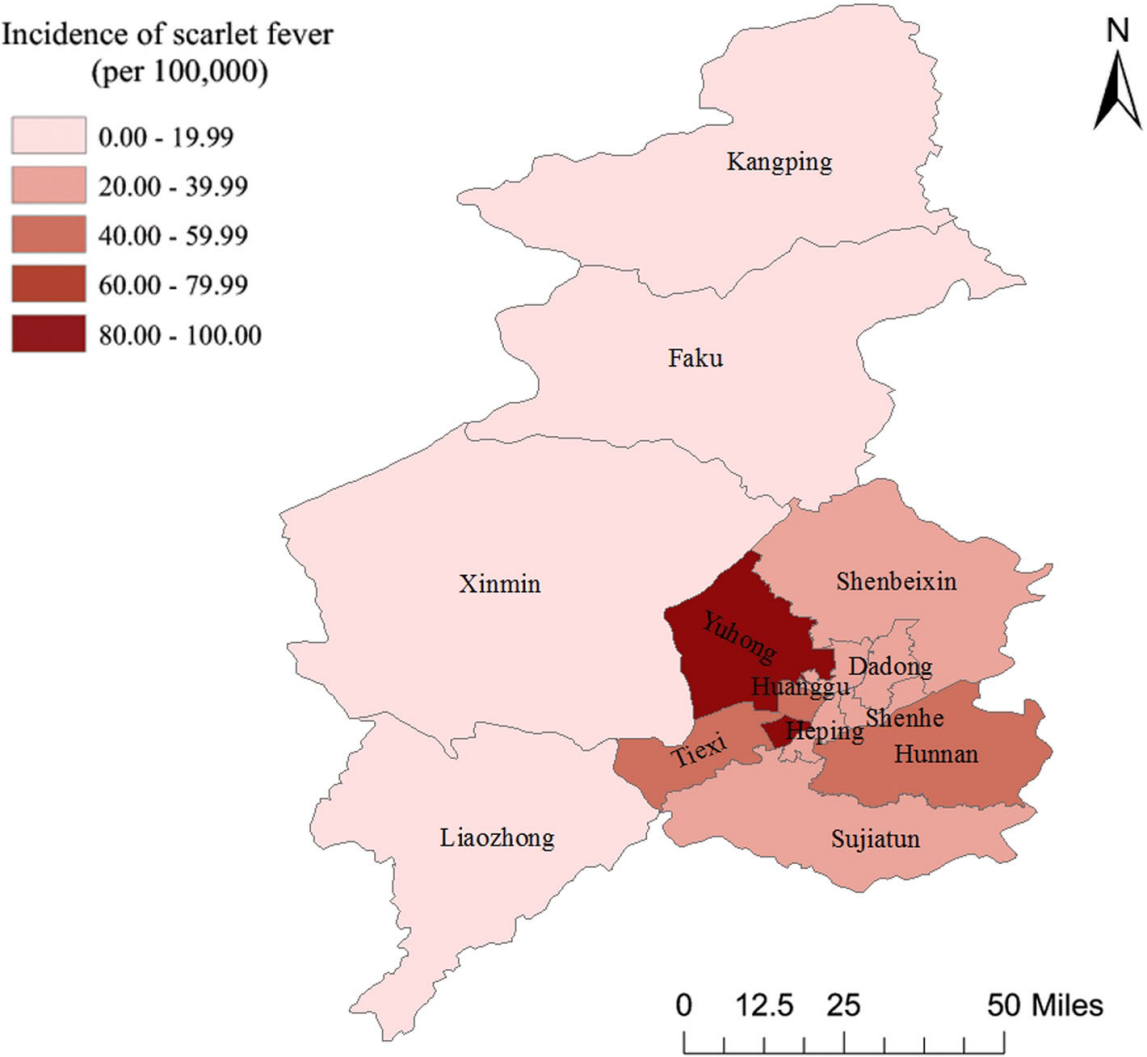
The spatial distribution of scarlet fever incidence in different districts in Shenyang in 2018. The annual incidence of scarlet fever per 100,000 residents in different districts in Shenyang in 2018 is shown in the map. The annual incidence of scarlet fever has a positive relationship with color darkness. This map was produced by ArcGIS software version 10.3 (ESRI, Redlands, CA, USA)

The distribution of scarlet fever incidence varied at the district level in Shenyang in 2018 showed that Yuhong district had the highest incidence, Hunnan and Tiexi district had relatively high incidence, the incidence of Faku district was the lowest. The incidence of scarlet fever in urban areas was significantly higher than that in rural areas (*p*<0.001) (Table 2 and Fig. 2).

**Table 2:** Incidence of scarlet fever in different districts/countries in Shenyang in 2018

Distrcts/countries	Cases	Population	Incidence
Urban areas			
Heping	227	698,304	32.51
Shenhe	172	712,589	24.14
Dadong	193	654,329	29.5
Huanggu	296	833,574	35.51
Tiexi	562	1,047,529	53.65
Sujiatun	130	424,689	30.61
Hunnan	162	388,078	41.74
Shenbeixin	99	326,243	30.35
Yuhong	353	410,975	85.89
Subtotal	2194	5,496,310	39.92
Rural areas			
Liaozhong	19	458,961	4.14
Kangping	7	342,918	2.04
Faku	11	439,224	2.5
Xinmin	83	670,825	12.37
Subtotal	120	1,911,928	6.28
X^2^			514.115
P			<0.001

Note**:** The chi-squared test done in Table 2 was testing for difference between urban areas and rural areas

### Spatial autocorrelation analysis and hotspot analysis

**Fig. 3:**
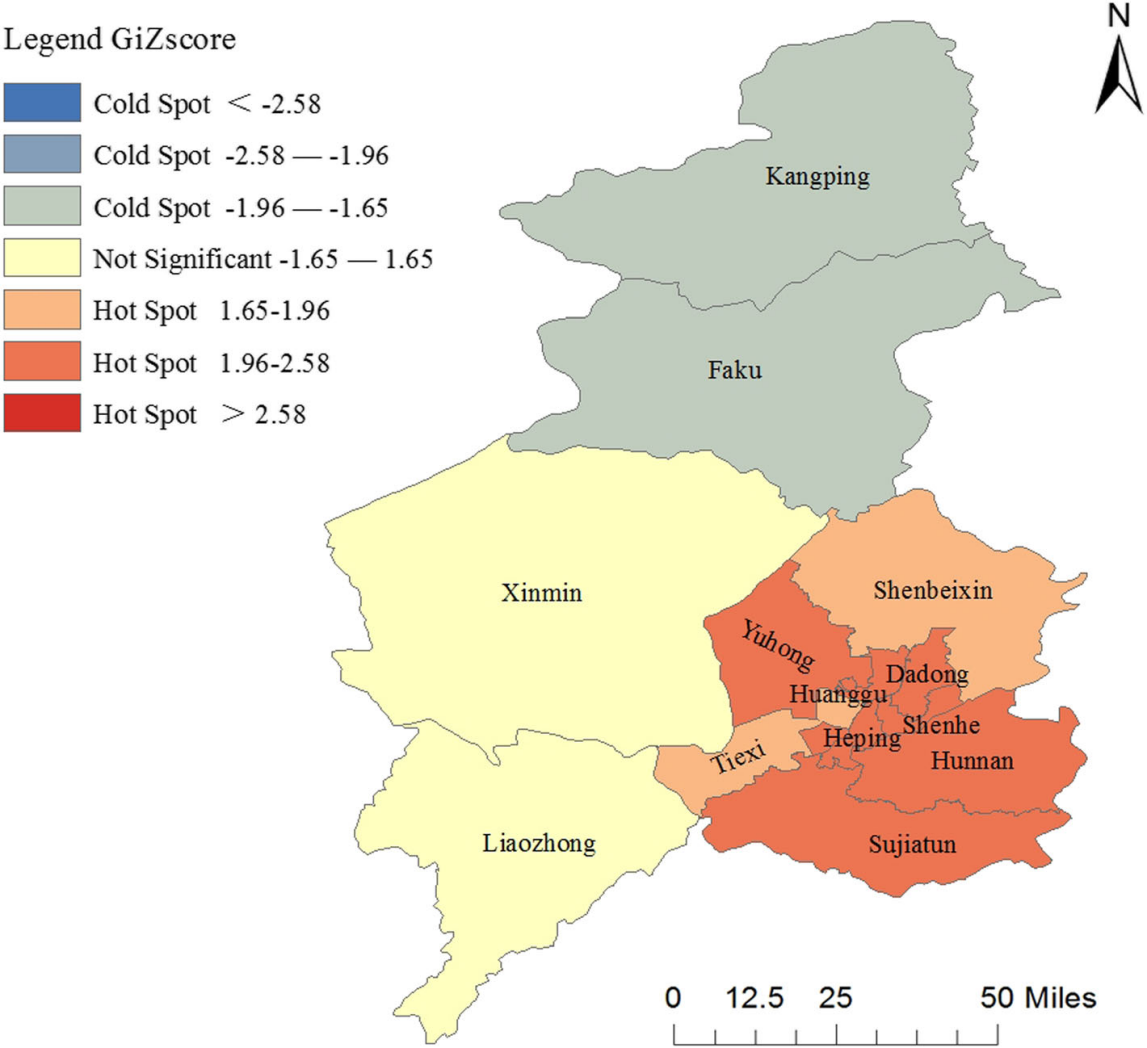
Hotspot clusters of scarlet fever incidence in Shenyang in 2018. Color depth indicates different Z-scores ranges. Districts with Z-scores > 2.58 or Z-scores < -2.58 were considered to be significant at 99% confidence level (*p* <0.01). Districts with Z-scores between 1.96 – 2.58 or Z-scores between -1.96 – -2.58 were considered to be significant at 95% confidence level (*p* <0.05). Districts with Z-scores between 1.65 – 1.96 or Z-scores between -1.65 – -1.96 were considered to be significant at 90% confidence level (*p* <.010)

Table 3 describes the result of spatial autocorrelation (Global Moran’s *I*) analysis and hotspot (Getis-Ord, Gi*) analysis in Shenyang. Spatial autocorrelation analysis showed that the incidence of scarlet fever was randomly distributed. The hotspot analysis demonstrates that hotspots (*p*<0.05) are located in seven districts, namely Heping, Hunnan, Shenhe, Huanggu, Yuhong, Dadong and Sujiatun district (Table 3 and Fig. 3).

**Table 3:** The results of the spatial autocorrelation and hotspot analysis of scarlet fever incidence in Shenyang in 2018

Autocorrelation Analysis			
Year	Moran *I*	*Z*-Score	*P*-Value
2018	0.099	1.741	0.082
Hot Spot Analysis			
Districts		Z-Score	P-Value
Kangping		−1.819	0.069
Faku		−1.819	0.069
Xinmin		1.298	0.194
Liaozhong		−0.048	0.962
Sujiatun		2.384	0.017
Dadong		2.533	0.011
Shenbeixin		1.742	0.081
Tiexi		1.851	0.064
Heping		2.533	0.011
Hunnan		2.533	0.011
Shenhe		2.533	0.011
Huanggu		2.533	0.011
Yuhong		2.283	0.022

## Discussion

Over the past decade, an exceptional upturn in the morbidity of scarlet fever has occurred in some Asian and European countries and areas, containing mainland China [20], Vietnam [21], Hong Kong [22], South Korea [23], Germany [24] and England [25] and the reasons remain unknown [26–29]. This is a worsening trend, especially in China where the ongoing resurgence in disease morbidity has exerted a marked influence on Chinese population since 2011 [20, 30]. To tackle this, understanding the epidemic characteristics of this disease may play a significant role in the allocation of limited health resource and the formulation of prevention and control strategies [31].

In this study, We found that the incidence of scarlet fever in Shenyang in 2018 is higher than that in Beijing [10] during 2005–2014 (14.25 per 100,000) and Jiangsu [9] during 2005–2015 (1.87 per 100,000),and is higher than the average annual incidence of the whole country [32] during 2003–2010 (1.58 per 100,000) and during 2011–2016 (4.14 per 100,000). We also found that the incidence of scarlet fever in 2018 was the highest since the outbreak of scarlet fever in 2011 in Shenyang [13, 14].

According to our study, the incidence of scarlet fever was higher among males than among females, which is consistent with other findings [9, 10, 33, 34]. The number of scarlet fever cases was the highest among children aged 3–11 years and accounted for 96.89%.The WHO and Public Health UK stated that a high-risk group of scarlet fever was among children 5–15 years old [35, 36] . In China, kindergarten education is at the age of 3–5 years and primary education is at the age of 6–11 years, so our study suggests that children in the kindergartens and the primary schools may be at high risk for scarlet fever.

Scarlet fever could occur throughout all the year, yet case notifications had a distinct seasonal distribution and showed double peak pattern in the year. There were fewer cases in February, and the number of cases increased sharply from March to June, the first peak occurred in June. The number of cases decreased from July to August and increased again between September and December, the second peak appeared in December, which is consistent with the findings of previous studies [9, 10, 31, 32]. In China, March–June and September–December are school days, and January–February and July–August are school holidays. It can be seen that the month in which the number of scarlet fever cases increases is the time when the children in the kindergartens and the primary school students are in school. The month in which the number of scarlet fever cases decrease is the time when the children in the kindergartens and the primary school students are on vacation. Prompting us that scarlet fever has obvious aggregation in kindergartens and primary schools. Since there is no scarlet fever vaccine at present, it may be helpful to suggest kindergartens and primary schools to implement the morning check system, epidemic reporting system and isolation measures. In addition, teachers and parents need to teach children to wash their hands frequently. Although previous studies have not consistently demonstrated direct transmission of GAS from fomites, proper maintenance of environmental hygiene remains a prudent measure to take [37]. Therefore, it is also suggested that kindergartens and schools improve environmental hygiene by disinfecting toys, railings and tables.

In our study, the disease mapping, spatial autocorrelation analysis and hot spot analysis were applied to depict the geographic distribution of scarlet fever incidence. The spatial distribution showed that scarlet fever cases were concentrated in urban areas with high population density, and the incidence of scarlet fever in urban areas was significantly higher than that in rural areas, consistent with the findings of Gehendra et al. [10] and this may suggest that the incidence of scarlet fever is related to population density. The autocorrelation analysis of Global Moran’s I value demonstrated that the spatial distribution of scarlet fever was randomly distributed in Shenyang in 2018. This meant that there was no autocorrelation of the spatial distribution of scarlet fever between districts in Shenyang. It indicated that there was neither positive correlation nor negative correlation between adjacent districts in the incidence, but a random distribution of high and low values with no rule to follow in Shenyang. However, hotspot analysis of Getis-Ord (Gi*) Z values revealed that the hotspot area with a high-high positive spatial association of scarlet fever incidence was located around the urban districts (Heping, Shenhe, Dadong, Huanggu, Sujiatun, Hunnan and Yuhong) and the coldspot area with a low-low positive spatial association of scarlet fever incidence was not found, which is consistent with the findings of Gehendra et al. [10] Prompting us that scarlet fever is easily to form aggregation in urban areas with high population density and convenient transportation which increased the risk of scarlet fever exposure [10]. These results remind us that prevention and control measures for scarlet fever should focus more on the hotspot areas.

In spite of the above findings, the limitations in our study should be considered. First, not all children aged 3–11 are enrolled in kindergarten or primary school and so further work is required to explore whether children in kindergarten or primary school are at higher risk of scarlet fever than children who are not in enrolled in school. Secondly, we did not analyze the reasons that the incidence is higher in Shenyang than in the rest of China, we will also conduct more studies to analyze the reasons for the higher incidence in Shenyang compared with other places in the future. Thirdly, our study confirmed once again that scarlet fever was more likely to occur in children, and once again emphasized the importance of strengthening prevention and control measures in kindergartens and primary schools, but our study provided no new information on risk factors, we will also do further work to study the risk factors of scarlet fever and provide more scientific evidence for the prevention and control of scarlet fever in the future.

## Conclusions

The monthly distribution of scarlet fever cases is obviously seasonal in Shenyang. The time distribution of scarlet fever is highly consistent with school and vacation time. Children aged 3–11 accounted for the vast majority of all scarlet fever cases. Prompting us that scarlet fever may have obvious aggregation in kindergartens and primary schools and it may be important to focus on the prevention and control of scarlet fever in kindergartens and primary schools. The incidence of scarlet fever in urban areas with dense population and convenient transportation is significantly higher than that in rural areas. Urban areas are the hotspots of scarlet fever which suggests that prevention and control measures for scarlet fever should focus more on the urban areas.

## Data Availability

The datasets used and analyzed during the current study is available from the corresponding author Huijie Chen (E-mail:chj1317@126.com) on reasonable request.

## Abbreviations

GAS: Group A Streptococcus
GIS: Geographic Information System
NIDRIS: Notifiable Infectious Diseases Reporting Information System
